# The impact of strain diversity and mixed infections on the evolution of resistance to *Bacillus thuringiensis*

**DOI:** 10.1098/rspb.2013.1497

**Published:** 2013-10-22

**Authors:** Ben Raymond, Denis J. Wright, Neil Crickmore, Michael B. Bonsall

**Affiliations:** 1 Mathematical Ecology Research Group, Department of Zoology, University of Oxford, Oxford OX1 3PS, UK; 2 Division of Ecology and Evolution, Department of Life Sciences, Faculty of Natural Sciences, Imperial College London, Silwood Park Campus, Ascot, Berkshire SL5 7PY, UK; 3 Department of Biochemistry, School of Life Sciences, University of Sussex, Falmer, Brighton BN1 9QG, UK; 4 St Peter's College, Oxford OX1 2DL, UK

**Keywords:** *Bt*, insecticide resistance, pest management, *Plutella xylostella*, synergism, transmission

## Abstract

Pesticide mixtures can reduce the rate at which insects evolve pesticide resistance. However, with live biopesticides such as the naturally abundant pathogen *Bacillus thuringiensis* (*Bt*), a range of additional biological considerations might affect the evolution of resistance. These can include ecological interactions in mixed infections, the different rates of transmission post-application and the impact of the native biodiversity on the frequency of mixed infections. Using multi-generation selection experiments, we tested how applications of single and mixed strains of *Bt* from diverse sources (natural isolates and biopesticides) affected the evolution of resistance in the diamondback moth, *Plutella xylostella,* to a focal strain. There was no significant difference in the rate of evolution of resistance between single and mixed-strain applications although the latter did result in lower insect populations. The relative survivorship of *Bt-*resistant genotypes was higher in the mixed-strain treatment, in part owing to elevated mortality of susceptible larvae in mixtures. Resistance evolved more quickly with treatments that contained natural isolates, and biological differences in transmission rate may have contributed to this. Our data indicate that the use of mixtures can have unexpected consequences on the fitness of resistant and susceptible insects.

## 1. Introduction

*Bacillus thuringiensis* (*Bt*) has become one of the most important sources of insect control agents in modern agriculture. Originally developed as a microbial pesticide in the 1930s, formulated *Bt* products that use bacterial spores and toxins are now valuable highly selective alternatives to synthetic insecticides in insect control, particularly in forestry, horticulture, disease-vector control, integrated pest management and organic agriculture [1]. Moreover, genetically modified (GM) crops expressing *Bt* toxins were planted in over 66 million ha in 2011 [2]. These plants are grown in 29 countries worldwide, with *Bt* genes being incorporated in the overwhelming majority of GM insect-resistant crops [2].

An ever-present threat to the economic sustainability of all pest control technologies is the evolution of resistance. Application of *Bt* microbial sprays has led to field resistance in two insect species, most commonly in the diamondback moth (*Plutella xylostella* L.) in intensive crucifer production [3]. Evolution of resistance to *Bt* transgenic crops has been reported for several insect pests [4–9]. The use of mixtures has a long history in resistance management for pesticides. Early theoretical work highlighted the benefits of mixtures relative to single insecticides [10], and predicted these benefits should increase with increasing dose and declining dominance of resistance [10–12]. The theory assumes that individuals carrying resistance genes for both pesticides are extremely rare, thus individuals carrying a resistance allele for one active ingredient will have little or no fitness advantage relative to susceptibles following application of high mixed doses. In practice, mixtures may not be universally beneficial because of the intense selection pressure for cross-resistance, and the financial cost and practical difficulty of ensuring equally high and equally persistent concentrations of two compounds [13]. For GM plants, these practical difficulties are less severe, because the expression of two *Bt* toxins at concentrations sufficient to kill monogenic resistant heterozygotes is practicable [12]. Experimental systems using *Brassica oleracea* expressing multiple *Bt* toxins have shown significant benefits in slowing the evolution of resistance relative to single toxin plants [14,15].

Resistance to *Bt* in the diamondback moth and other Lepidoptera is most commonly associated with mutations in the insect that prevent the Cry toxins from binding to the gut epithelium [16]. Although Cry toxins are the primary virulence factors, and the main selective agents for resistance, for live microbial sprays other factors may influence the evolution of resistance that have not been fully addressed. First, the ecological interactions of multiple strains in mixed infection can have important consequences: strains can interact synergistically to increase mortality [17], or compete antagonistically and reduce host mortality [18]. These interactions may have consequences for the relative fitness of resistant insects. Second, microbial sprays cause mortality in conjunction with a naturally occurring bacterial community. This natural population of *Bt* is extremely widespread and can be locally highly abundant, so that diverse populations of bacteria can persist patchily on single leaves shortly after the application of biopesticides [19,20]. The substantial impact of this community on insect resistance can be inferred from the relatively high frequency of *Bt* resistance alleles (10^−1^ to 10^−3^) in folivorous pest populations prior to the extensive exposure to commercial *Bt* products [21]. Applications of *Bt* microbial sprays can affect the diversity and structure of these bacterial communities [20]. While *Bt* sprays may persist for only a short time on leaves, their impact on microbial communities in the soil may be longer lasting [22]. If *Bt* sprays reduce the diversity of naturally occurring strains, then this may compound issues of the evolution of resistance.

Perfect execution of the recommendations of the theoretical models of mixed insecticides can have predictable consequences. However, these recommendations may often be set aside, either for short-term practicalities, or because growers are desperate or uninformed and combine products ad hoc [23,24]. In these scenarios, the biological properties of biopesticides and their ecological interactions are likely to be important. Here, we aimed to understand how altering the diversity of *Bt* strains affected the evolution of resistance, and we sought to do this with (i) biopesticide strains that have been combined in mixed-spray applications, and (ii) with a range of wild-type strains that might co-occur in the field in a multi-generation selection experiment. We used the diamondback moth, *Plutella xylostella* as our target insect and measured the changes in resistance to a focal *Bt* strain (*Bt* subp. *kurstaki* HD1) [*Btk*]. Following the results of our selection experiment, we explore hypotheses that could explain the poor efficacy of diverse applications in slowing the evolution of resistance and the different consequences of applying strains of wild-type and biopesticidal origin.

## 2. Methods

### (a) Bt strains and insects

The *Btk-*resistant strain of diamondback moth Kar/UK_6_/Newc and its near isogenic susceptible counterpart UK/Newc were produced as described previously [25]. Resistance was maintained by regular selection using 50–100 µg ml^−1^ of a *Btk* HD1 product, DiPel DF (Valent Biosciences) [26]. Throughout this study, insects were fed on Chinese cabbage, *Brassica pekinensis* (Lour.) Rupr. cv ‘one kilo, S.B’. Both biopesticides, DiPel DF and XenTari, a product derived from *B. thuringiensis aizawai* (Valent Biosciences) were sourced from dry formulated products (table 1). *Bt* subsp. *entomocidus* HD9/BGSC 4I4 originated from the Bacillus Genetic Stock Centre (Department of Biochemistry, Ohio State University). *Bt* isolates A2m21 (*B. thuringiensis kurstaki*) and C3s3 (*Bacillus weihenstephanensis*) were collected in the Silwood Park campus (Ascot, UK) and donated by Ellis and co-workers [29]. Dt 7.1.o (also *Bt kurstaki*) was collected in the UK by B.R. The principal selective agents in these bacteria are the Cry toxins. DiPel DF contains Cry 1Aa, Cry1Ab, Cry1Ac and Cry2Aa [30], XenTari contains Cry1Aa, Cry1Ab, Cry1C and Cry1Da toxins [31], the toxins produced by *Bt* subsp. *entomocidus* HD9 have not been described previously. Toxin profiles of all strains were examined using SDS–PAGE (see the electronic supplementary material, figure S2). The HD9 toxin complement differs from that of the XenTari and *kurstaki* strains and contains a higher molecular weight toxin consistent with previous reports that this subspecies encodes a Cry1B toxin [32].

**Table 1.** Pathogenicity of strains to *Btk-*resistant and susceptible diamondback moth in leaf dip bioassays. (Data are LC_50_s with 95% CLs (ln spores ml^−1^). Superscripts indicate whether LC_50_s were significantly different among genotypes. RR is the resistance ratio—the untransformed LC_50_ of resistant insects ÷ LC_50_ of susceptibles. *D*_LC_ is the dominance of resistance at the LC_50_ concentration, for log-transformed data this was calculated (LC_F1_-LC_susc._)/(LC_res._-LC_susc._), with a maximum value of 1.0 [27]. Data for the C3s3 *B. weihenstephanensis* strain are not shown, as this strain did not kill any insects at any concentration. Predicted LC_50_s of the mixtures are based on the weighted harmonic means of the LC_50_s of their components [28].)

strain/product	subspecies	LC_50_ for each larval genotype					
		susceptible	F_1_ (resistant father)	F_1_ (resistant mother)	resistant	RR	*D*_LC_
Dt 7.1.o	*B. t. kurstaki*	9.37^a^ (8.73, 10.0)	11.6^b^ (10.7, 12.45)	12.0^b^ (11.4, 12.6)	14.3^c^ (13.6, 15.1)	144	0.49
DiPel DF	*B. t. kurstaki*	8.51^a^ (7.73, 9.27)	11.4^b^ (10.6, 12.1)	11.1^b^ (10.5, 11.7)	16.2^c^ (14.0, 18.4)	2230	0.36
A2m21	*B. t. kurstaki*	9.33^a^ (8.93, 9.73)	10.2^a^ (9.69, 10.7)	9.87^a^ (9.41, 10.3)	14.8^b^ (14.0, 15.6)	244	0.13
XenTari	*B. t. aizawai*	11.1^a^ (10.7, 11.6)	12.2^a^ (11.4, 12.9)	11.0^a^ (10.3, 11.7)	12.1^a^ (11.5, 12.7)	2.63	n.a.
BGSC 4I4	*B. t. entomocidus*	10.5^a^ (10.1, 10.9)	11.6^b^ (11.1, 12.0)	n.a.	11.3^b^ (10.9, 11.6)	2.13	1.0
selection treatment		predicted LC_50_s, RR and D_LC_					
DiPel DF + XenTari		9.63	11.4		13.9	68.0	0.41
‘six strain’		11.6	13.4		16.2	97.5	0.40

All *B. thuringiensis* field isolates were identified by sequencing of the flagellin *Bthag* gene [33,34]. Dt 7.1.o and C3s3 have also been characterized using the *Bacillus cereus* group multilocus sequence typing scheme (http://pubmlst.org/bcereus/) [35], and sequence types recorded under strains 523 and 536, respectively. Spores and toxins for all wild-type strains were produced for each passage on *B. cereus*-specific agar (Oxoid, UK) as described previously [36] after being refreshed from glycerol stocks. Formulated biopesticides were sourced from single batches throughout selection. Wild-type spore/toxin suspensions were stored at 10°C in sterile saline (0.75% NaCl) and used within two weeks.

### (b) Susceptibility bioassays

Single-strain assays of second-instar larvae used established leaf dip bioassays [37], with five concentrations and 1.9 × 10^7^ spores ml^−1^ as the highest concentration, spores were enumerated by haemocytometer counts. Heterozygote insects (F_1_ crosses of Kar/UK_6_/Newc and UK/Newc) were produced via ‘mass crossing’ of approximately 150 adults, with individual mating cages for each reciprocal cross. Parents were derived from susceptible, and recently selected resistant insects that were stored singly in 30 mm Petri dishes on pupation (to prevent uncontrolled mating); pupae were checked daily and sexed on emergence before being transferred to mating cages.

### (c) Selection experiment and mixed-strain assays

**Figure 1.**
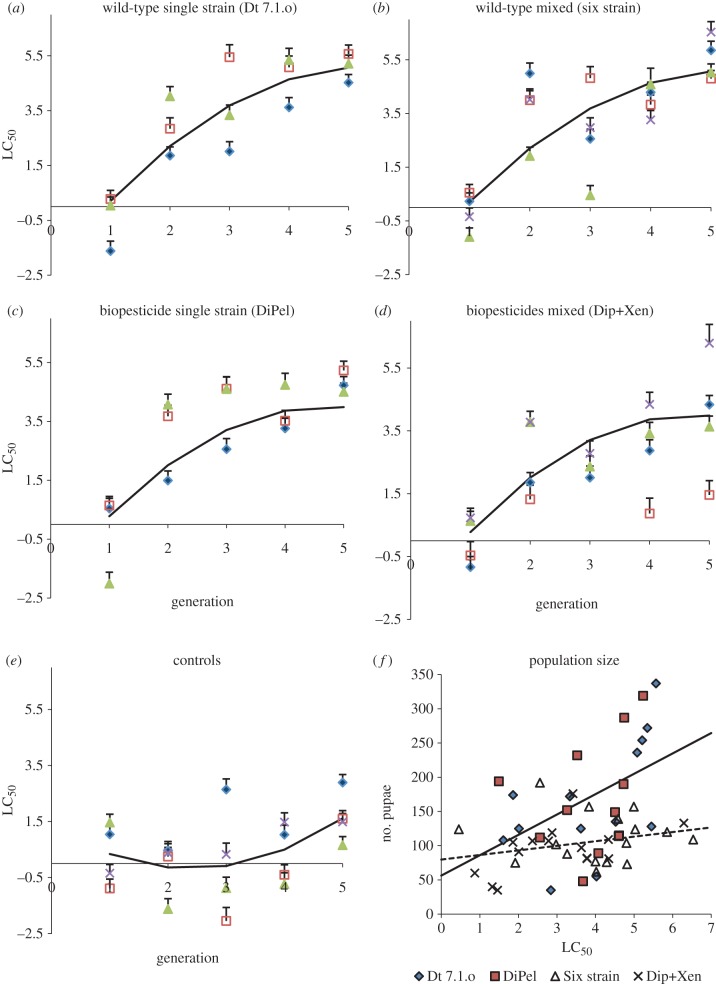
Evolution of resistance to *Btk* by selection with single- and mixed-strain infections of varying biological origin over five generations of selection in 18 independent selection cages. Selection treatments were (*a*) the wild-type *B. t. kustaki* Dt 7.1.o; (*b*) the six strain treatment; (*c*) DiPel DF, (*d*) the biopesticides DiPel DF and XenTari in a mixture, and (*e*) the unselected controls. Data (*a*–*e*) are LC_50_s + SEs for DiPel DF concentrations (natural logarithm transformations of μg ml^−1^) based on independent bioassays of each cage at each generation. Different symbols within each subfigure represent different cages, whereas lines represent fitted mixed models for controls, pooled wild-type treatments (Dt 7.1.o wild-type, six strain) and pooled biopesticide treatments (DiPel, DiPel and XenTari). (*f*) The relationship between population size (number of pupae surviving the previous generation) and LC_50_ for selected cages in generations 2–5; the solid line is the fitted model for the single-strain treatments, the dashed line represents the mixed-strain treatments.

Selection experiments were initiated with 100 UK/Newc pupae and eight F_2_ pupae from a Kar/UK_6_/Newc X UK/Newc cross and run in culture cages (1 × 0.75 × 0.55 m) at 20–22°C. Adults laid eggs on three Chinese cabbage plants; when leaf mines produced by first-instar larvae appeared, one plant was removed from each cage for subsequent bioassays, whereas the remaining two plants received experimental sprays. We imposed four *Bt* treatments and one control (water and Triton X-100 × 100 µl l^−1^) with four replicate cages per treatment and imposed five generations of selection. Bioassays conducted after one generation of selection revealed little variation in initial conditions (figure 1). In each treatment, we maintained a constant total spore concentration of 3.8 × 10^6^ spores ml^−1^, we imposed two single-strain treatments (DiPel DF and Dt 7.1.o) and two mixtures, this concentration was expected to produce more than 90% mortality in the susceptible population based on the single-strain leaf dip assays (table 1). The biopesticide mixture used equal concentrations of DiPel DF and XenTari, whereas the six strain mixture used equal concentrations of all strains listed in table 1, plus the *B. weihenstephensis*. Plants were sprayed with 40–50 ml of the appropriate spore/toxin suspension, until run-off. After one week of feeding, survivors were predominantly fourth instar, and two additional unsprayed plants were added to each cage in order to supply sufficient leaf material for successful pupation; larval populations in control cages were culled by transferring two leaves from each of the original plants onto each fresh plant. Surviving pupae were collected over the next 4–5 days, bulked by storing at 10°C and counted before initiating the next generation. Leaf dip assays to assess resistance during the experiment used third-instar larvae and formulated DiPel DF, as described previously [37].

Mixed-strain assays used plants sprayed in the same manner as in the selection experiment; 3 × 50 mm leaf discs from leaves of varying age were cut from each sprayed plant and placed in 90 mm Petri dishes with 25 early second-instar larvae. Each treatment was tested using three insect genotypes (Kar/UK_6_/Newc, UK/Newc and their F_1_ crosses) and was replicated on six different plants. Calculation of the effective dominance of resistance (*D*_ML_) followed standard methods for single doses [27]. Antagonistic or synergistic interactions on mortality in a mixed infection occur when observed mortality is lesser or greater than expected based on the independent contribution of the two components [28]. This can be assessed by comparing mortality in simultaneous assays of mixtures and each mixture component, in which the total dose is standardized [18,38,39]. If mortality in the mixture exceeds that of the most potent component of the mix, then the components are interacting synergistically; if mortality is depressed relative to that of the least potent component, then strains are interacting antagonistically. By conducting these assays with the treatments used in the selection experiment, we were able to test for synergism by comparing mortality in the mixtures with that in treatments with the most potent components (DiPel DF and Dt 7.1.o).

### (d) Transmission experiment

We assayed the infectivity of cadavers produced after exposure to our four selection regimes in mini-plants (three cabbage leaves in water) setup in the same environmental conditions as the original selection experiment. We attached two fresh cadavers (approx. one week old) to each leaf with a sterile toothpick and circled each inoculating cadaver with a red permanent marker. Twenty four uninfected second-instar larvae were then placed on each mini-plant, and the presence of additional cadavers recorded regularly until day 8. *Bt*-killed cadavers were identified by extensive melanization. We used a factorial design with four bacterial mixtures (and one control), crossed with two uninfected insect genotypes (Kar/UK_6_/Newc, UK/Newc) and 12 replicates per treatment.

### (e) Statistical analysis

Mortality data were analysed with generalized linear models (GLMs) using a logit link function and binomial errors in R v. 2.6.2 (http://www.r-project.org). *F*-tests using the function ‘quasi-binomial’ were applied to correct for over-dispersion where appropriate [40]. Estimates of lethal concentration resulting in 50% mortality (LC_50_) and their standard errors were calculated according to individually fitted analyses of deviance [41]. The selection experiment was analysed using arc-sine-transformed proportions in maximum-likelihood mixed model ANOVA with selection treatment, log bioassay concentration and generation of selection as main (fixed) factors. Heterogeneity in response to selection between independent replicates (selection cages) was accounted for by incorporating cage as a random effect. Comparisons of models with different fixed effect structures using likelihood ratio tests following maximum-likelihood model fitting. Final model fitting and model assumptions were checked with graphical analyses [40]. A complementary analysis of the selection experiment used cage-level LC_50_s calculated after fitting using independent binomial GLMs at each generation, with common slopes for all cages within each generation. Two cages, in which diamondback moth populations went extinct before the end of the experiment, were excluded from the LC_50_ analysis.

## 3. Results

### (a) Relative resistance in single-strain assays

We assayed susceptibility of *Btk*-resistant (Kar/UK_6_/Newc) and *Btk* susceptible (UK/Newc) diamondback moth larvae, as well as their reciprocal F_1_ hybrids, to each of the experimental strains using a leaf dip bioassay. High levels of resistance were observed towards the three *kurstaki* strains and, in each case, a pattern of incomplete dominance was observed (table 1 and raw data shown in the electronic supplementary material, figure S1). The greatest level of resistance was found against the DiPel biopesticide-derived strain of *Bt kurstaki*. Low, but dominant cross-resistance was found to the *Bt* subsp. *entomocidus*, whereas cross-resistance to *Bt* subsp. *aizawai* was not significant in this study. The *Bt weihenstepanensis* strain was not virulent to any of the *P. xylostella* genotypes.

### (b) Selection experiment

We investigated the rate of increase of resistance to our focal *Btk* strain following exposure to single, or mixed, applications of *Bt* of varied biological origins. Inspection of LC_50_s (figure 1*a–e*) indicates that resistance increased in all experimental cages (where moth populations remained viable), with the exception of one cage in the DiPel + XenTari treatment. Resistance remained relatively constant in the control cages, although there was some variation between cages and between generations. Resistance increased more quickly in the cages with treatments containing wild-type strains (figure 1*a,b*). A mixed model analysis of LC_50_s found no significant difference in the rate of change of LC_50_s between single- and mixed-strain selection treatments (likelihood ratio = 4.26, d.f. = 3, *p* = 0.2348). Calculating the response to selection based on changes in LC_50_ also indicated that the response to selection was similar across all treatments, bar controls (see the electronic supplementary material, table S2).

An additional analysis was performed using a mixed model analysis of arc-sine-transformed mortality data. Because this incorporated all the bioassay data across five generations, rather than rely on a single summary statistic (LC_50_), this analysis should be more sensitive. Model simplification as a means of significance testing, i.e. the sequential removal of treatments or factor levels in maximum-likelihood fitted models followed by ANOVA based on likelihood ratios [37]. Raw bioassay data and fitted mixed models are displayed in the electronic supplementary material, figure S3 and table S3. This analysis confirmed that the use of single- or mixed-strain applications *per se* had no effect on the evolution of resistance. Thus, selection experiments that included wild-type strains (Dt 7.1.o and the six strain treatment) resulted in similar changes in *Btk* resistance during the selection experiment, regardless of the large differences in strain diversity (likelihood ratio = 2.49, *p* = 0.48; electronic supplementary material, table S3). Similarly, both treatments using biopesticide-derived strains (Dipel, DiPel + Xentari) caused similar changes in resistance (likelihood ratio = 2.07, *p* = 0.56; electronic supplementary material, table S3). What was demonstrated however was that the biopesticide and wild-type strain selection treatments behaved differently, with resistance to *Btk* being highest in the wild-type treatments at the end of selection (likelihood ratio = 16.0, *p* = 0.0012; electronic supplementary material, table S3). Formally, wild-type-selected cages became less responsive to increasing concentrations as the selection experiment went on (log concentration × generation × treatment interaction, *t* = −2.01, *p* = 0.445; electronic supplementary material, figure S3).

Despite not affecting resistance any more than single-strain applications, mixed applications of diverse *Bt* strains did have benefits in terms of maintaining lower insect population sizes as the selection experiment progressed. In the single-strain treatments, increasing resistance led to higher insect population size, measured as the numbers of pupal survivors at the end of each generation (treatment × generation interaction: likelihood ratio = 4.22, d.f. = 1, *p* = 0.04). By contrast, increasing *Btk* resistance did not lead to an increase in population size in cages exposed to diverse strains (post hoc test for significant positive slope *t* = 0.99, d.f. = 39, *p* = 0.33). Here then, strain diversity, but not biological origin was important, as both single-strain treatments had similar effects on population dynamics, as did both mixed-strain treatments (likelihood ratio = 2.05, d.f. = 4, *p* = 0.73).

### (c) Transmission experiment

**Figure 2.**
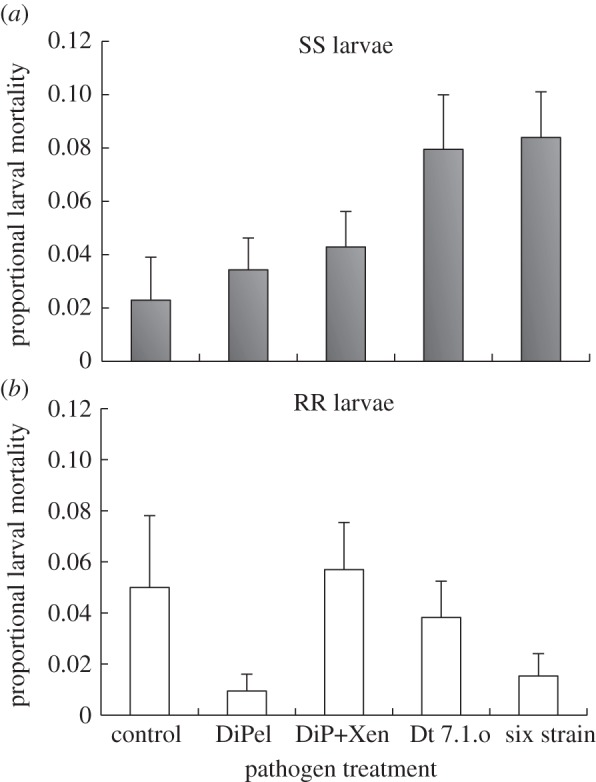
(*a*,*b*) The infectivity of cadavers to larvae of diamondback moth on experimental mini-plants over 8 days. Data are plotted according to the genotype of larvae exposed to infection: homozygous susceptible (SS) and homozygous resistant (RR). Cadavers were produced according to the treatments used in the selection experiment: DiPel (DiPel only); DiP+Xen (DiPel XenTari mixture); Dt 7.1.o (wild-type *Btk*); six strain (diverse mixture of six strains). Data are means + s.e., with standard error calculated according to normal approximation to the binomial.

**Figure 3.**
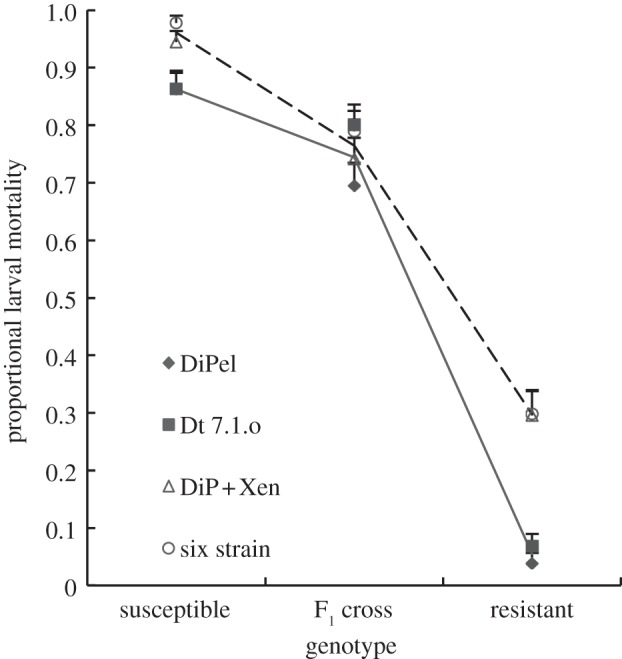
Mortality and dominance of resistance after exposure to the single- and mixed-strain applications used in the whole plant selection experiment*.* Data are mean mortality + s.e.s calculated from six independent plants (*N* ≅ 130 insects in total per treatment). The lines are the minimal adequate statistical models fitted to the observed mortality data: the solid line represents the mixed-strain treatments, the dashed lines the single-strain treatments. Open symbols are single-strain data, filled symbols are mixtures containing wild-type bacteria.

In order to investigate the accelerated evolution of resistance in response to wild-type strains, we tested the hypothesis that cadavers produced by wild-type strains were more infectious than those produced by biopesticide-derived strains. In support of this hypothesis, we found that cadavers produced by sprays incorporating wild-types were more infectious to susceptible larvae than cadavers killed by biopesticides (spray treatment ×genotype interaction, *χ*_4_^2^ = 11.4, *p* = 0.023; figure 2). However, *Btk-*resistant larvae did not appear to be susceptible to infection from cadavers, as mortality rates were indistinguishable from controls (figure 2; *post hoc* contrasts indicated no significant difference in comparison with controls). Larval mortality rates for both the wild-type treatments (Dt 7.1.o and the six strain treatment) were very similar, and these treatments could be pooled without significant loss of deviance (*χ*_2_^2^ = 2.01, *p* = 0.37; figure 3). The genotype of the insect producing the original cadaver had no impact on transmission (main effect *χ*^2^_2_ = 0.06, *p* = 0.97).

### (d) Effects of mixed sprays on mortality and relative survivorship of *Btk*-resistant insects

We investigated the consequence of our selection regimes on the relative survivorship of resistant and susceptible insects in sprayed plant assays. Both treatment regime and larval genotype affected mortality (figure 3; interaction *F*_4,63_ = 4.30, *p* = 0.0039; treatment main effect *F*_2,67_ = 194, 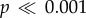; genotype *F*_2,69_ = 8.89, *p* = 0.0004). The statistical model could be simplified by pooling the single-strain treatments (DiPel and Dt 7.1.o) into one group and the mixed-strain treatments (DiPel + XenTari and six strain mix) into another without loss of explanatory power (*F*_3,63_ = 2.99, *p* = 0.52). Mortality on whole sprayed plants was lower than predicted from the same concentration of spores in the leaf dip assays.

The *Btk* strains (DiPel, Dt 7.1.o, A2m21) were the most potent components of the mixtures for *susceptible* insects (table 1, note lower LC_50_s). Thus, the mixed-strain applications were effectively diluting the more potent strains with less potent ones. A null hypothesis of no interaction among strains in mixed infections would therefore predict lower mortality for susceptible insects in mixtures relative to the most potent component in this assay, as we controlled for total overall concentration. We found the opposite pattern: mixtures imposed more mortality on susceptible insects than single-strain treatments (*F*_1,22_ = 8.79, *p =* 0.0071, one-tailed *post hoc* comparison, difference estimate = 1.35, *t* = 1.35, s.e. = 0.49, *p =* 0.0062; figure 3), a pattern consistent with synergistic interactions among the different *Bt* strains.

An important effect of the elevated mortality of susceptible insects in mixtures was an increase in the relative survivorship of resistant insects. The relative survivorship of the susceptible, F_1_ hybrid and resistant genotypes was 0.14, 0.27 and 1.0, respectively, in the single-strain sprays and 0.06, 0.34 and 1.0 in the mixed-strain sprays, based on the parameter estimates for the GLM of observed mortality above. The effective dominance (*D*_ML_) of resistance was higher in the mixed-strain sprays (0.30) relative to single-strain sprays (0.15), a result that was not expected based on the patterns of dominance in our single-strain leaf dip bioassays (table 1).

## 4. Discussion

We expected that lower pathogen diversity, which is one possible environmental consequence of biopesticide application [20], would increase the rate of evolution of resistance. In this study, the evolution of resistance was insensitive to pathogen diversity, although more diverse bacterial infections did lead to lower population sizes during the selection experiment. The biological and ecological characteristics of our experimental strains had important and novel consequences for the evolution of resistance. When strains were bioassayed individually in resistant and susceptible insects, the relative resistance ratio of insects exposed to DiPel was more than 10-fold greater than that for the wild-type *Btk* Dt 7.1.o (table 1), suggesting that there would be more intense selection pressure for resistance using DiPel. We observed the opposite: increased rates of evolution of *Btk* resistance with selection using wild-type strains (figure 1 and electronic supplementary material, figure S3). Our experimental design made cadaver to larva transmission possible, and we found that cadavers produced by wild-type strains were more infectious than those produced by biopesticide-derived strains. We would expect more infectious cadavers to lead to increased opportunities for additional rounds of infection and therefore potentially more intense selection pressure. Horizontal transmission directly from DiPel*-*killed cadavers has been shown to be weak [42]. Nevertheless, recently isolated strains can produce more infectious cadavers than biopesticides, which can be ascribed to increased spore production per cadaver [43].

The results here agree with previous work showing that mixtures of *Bt* strains did not greatly slow the evolution of resistance [44], but differ from results with GM plants showing that resistance evolved slower to plants producing two toxins than to plants producing one toxin [14,15]. With the GM plant experiments, the two toxins were chosen because little or no cross-resistance occurs between them, so combining them is expected to double the diversity of mortality factors [14,15]. By contrast, some of the strains tested here share the same toxins (e.g. Cry1Aa and Cry1Ab occur in both DiPel and XenTari) or have closely related toxins between which cross-resistance does occur (e.g. Cry1Ac in DiPel and Cry1Ab in XenTari). Because of this overlap in toxins between strains, mixing two strains does not necessarily double the diversity of mortality factors. Without more information about the relative potency and relative abundance of the toxins in each strain, we cannot determine how much the diversity of mortality factors was increased by combining two or more multi-toxin strains. There were also differences between our study and previous experiments using GM plants: we did not explicitly include refugia, i.e. unsprayed plants in selection cages, although spray application will lead to substantial variation in concentrations within plants. We also did not investigate cross-resistance or the evolution of resistance at two loci. Concentrations of spores and toxins in our experiment were also not high enough to impose fully recessive resistance, but chosen to allow us to determine how biological interactions among strains might affect mortality. Notably, the response to selection with mixtures was quite diverse in the DiPel + XenTari treatment, with one out of four cages showing little evolution of resistance, whereas another cage showing very rapid appearance of resistance (figure 1). Average responses, however, indicated that there were few benefits to be gained from using this mixture to delay the evolution of resistance.

Genes of large effect are the most common mode of *Bt* resistance [21]. Effective resistance management to pesticides therefore depends on altering fitness of initially rare alleles that are generally present in heterozygotes. Following this ‘single major gene’ model of *Bt* resistance, the relative fitness of heterozygotes to susceptible insects determines initial rate of increase. This, in turn, is affected by frequency of exposure (or proportion of population under selection) [45] and by dose, which determines levels of dominance [27]. High doses, which lead to increasingly recessive resistance, have featured in resistance management advice for many years [10–12,46,47], and failure to maintain these high doses in GM crops expressing *Bt* toxin has been associated with subsequent resistance problems in several cases [5,6].

In this study, we observed elevated mortality of susceptible insects in mixed sprays. This was unexpected. The sources of diverse toxins that could effectively target *Btk-*resistant insects in the mixed applications were the *Bt aizawai* and *Bt entomocidus* strains (table 1). Because these strains tended to be less potent for *Btk* susceptible insects and more potent in *Btk-*resistant insects, we expected mixtures to reduce the mortality of susceptible insects and increase the mortality of *Btk-*resistant strains (table 1 and electronic supplementary material, figure S1). The LC_50_s of F_1_ heterozygotes were relatively similar for all strains bar A2m21, so we expected similar mortality for heterozygotes in mixtures and single-strain applications, at least for the biopesticide mix. Applying Tabashnik's method for predicting LC_50_s of mixtures [28] to the data obtained from the leaf dip bioassays (table 1) also did not predict that the mixtures used in the whole plant assay would lead to increased mortality. When we assessed the effect of the various treatments under the conditions used in our selection experiment, we found that while the resistant and heterozygotes insects behaved as expected (based upon their individual effects in the leaf dip assay), there was a higher than expected mortality of susceptible insects in mixed sprays relative to single-strain sprays (figure 3). There are several possible explanations for the higher mortality of susceptibles in mixed sprays. First, there is a possibility of synergistic interactions among the different strains and/or toxins produced by distinct strains [48,49]. Second, given that we controlled for spore concentrations, not doses, there is the possibility that differences in larval behaviour in mixtures and single-strain applications affected ingested doses. Spraying or dipping of leaves can result in heterogeneous distributions of spores and toxins, and behavioural avoidance of spores and toxin is well documented [50,51], behavioural differences can also exist between resistant and susceptible genotypes [50]. Alternatively, given that invertebrates can upregulate diverse genes in response to *Bt* toxicity [52], it is possible that the instar initially exposed to spores and toxins on plants (neonates on whole plants, second instars in bioassays) can affect the level of mortality.

In summary, the impact of biopesticides on reducing native strain diversity is not a major issue for resistance management, because decreased diversity does not appear to exacerbate the evolution of resistance. Increased background population densities of spores from repeated spray applications may conceivably lead to longer-term selection pressure. However, field experiments have shown that single applications of *Bt* have relatively transient effects on spore density in soil and on plant leaves [20]. The effect of insects themselves may have more durable effects on *Bt* populations in agro-ecosystems [20]. Increasing strain diversity via the application of multiple biopesticides can do two things: it can improve pest population control, as we found here, and it can improve resistance management. In some circumstances, however, these effects can be mutually exclusive. Here, we show that increased mortality may be one consequence of mixed-spray applications. Furthermore, when effective doses are not high, multiple active compounds have the potential to act synergistically. If synergistic interactions act only on the susceptible genotype, while interactions are additive for resistant individuals, then mixtures at least have the potential to increase the relative fitness of resistant individuals, as has been shown for resistance to fungicides [53].
